# Sericin Enhances the Bioperformance of Collagen-Based Matrices Preseeded with Human-Adipose Derived Stem Cells (hADSCs)

**DOI:** 10.3390/ijms14011870

**Published:** 2013-01-16

**Authors:** Sorina Dinescu, Bianca Galateanu, Madalina Albu, Anisoara Cimpean, Anca Dinischiotu, Marieta Costache

**Affiliations:** 1 Department of Biochemistry and Molecular Biology, University of Bucharest, 91-95 Splaiul Independentei, Bucharest 050095, Romania; 2 Collagen Department, Leather and Footwear Research Institute, 93, Ion Minulescu, Bucharest 031215, Romania

**Keywords:** collagen, sericin, hADSC, biocompatibility, adipogenesis, proliferation, porous biomatrix, adipose tissue engineering

## Abstract

Current clinical strategies for adipose tissue engineering (ATE), including autologous fat implants or the use of synthetic surrogates, not only are failing in the long term, but also can’t face the latest requirements regarding the aesthetic restoration of the resulted imperfections. In this context, modern strategies in current ATE applications are based on the implantation of 3D cell-scaffold bioconstructs, designed for prospective achievement of *in situ* functional *de novo* tissue. Thus, in this paper, we reported for the first time the evaluation of a spongious 60% collagen and 40% sericin scaffold preseeded with human adipose-derived stem cells (hADSCs) in terms of biocompatibility and adipogenic potential *in vitro*. We showed that the addition of the sticky protein sericin in the composition of a classical collagen sponge enhanced the adhesion and also the proliferation rate of the seeded cells, thus improving the biocompatibility of the novel scaffold. In addition, sericin stimulated PPARγ2 overexpression, triggering a subsequent upregulated expression profile of FAS, aP2 and perilipin adipogenic markers. These features, together with the already known sericin stimulatory potential on cellular collagen production, promote collagen-sericin biomatrix as a good candidate for soft tissue reconstruction and wound healing applications.

## 1. Introduction

In the field of tissue engineering, besides the reconstruction of the functional tissue, a modern requirement is also the aesthetic restoration of the imperfections resulting from traumatic injury, tumor resection and congenital defects [1]. Current clinical strategies for adipose tissue engineering (ATE) include the use of autologous fat implants, which are considered to be the ideal filling material in terms of biocompatibility, immune response and avoidance of graft rejection [2]. However, adipose tissue transplantation yields unpredictable results, due to varying degrees of graft resorption over time (40%–60% volume loss) and lack of sufficient revascularization [3,4].

The alternative use of synthetic surrogates (Teflon, silicone implants) or allogenic materials, like bovine collagen, have the advantage of endless supply, but clinical experiences revealed various deficiencies, such as rupture, capsular contracture, dislocation, suboptimal biocompatibility of the implants and allergic reactions [5,6].

Modern strategies in current ATE applications involve the design of 3D cell-scaffold bioconstructs obtained by preseeding the scaffold with undifferentiated cells. In order to achieve *in situ* functional *de novo* tissue, the embedded cells are committed towards the adipogenic lineage by subjecting the bioconstructs to *in vitro* adipogenic conditions. Subsequently, the engineered tissue is expected to be structurally, mechanically and functionally integrated to the implantation site. Overall, the most important feature of this modern strategy is the achievement of a long-term and predictable clinical application result ensured by the control of the scaffold’s composition, implanted cell number and the differentiation status and kinetics.

Regarding the cellular component of the bioconstructs, attempts to engineer adipose tissue have involved the use of preadipocytes and adipocytes as the base cell source. Increased interest surrounding the research and development of stem cells as a source of cells for tissue engineering has led to novel ATE strategies [1]. Human adipose derived stem cells (hADSCs) are currently a viable source of mesenchymal-like stem cells for ATE applications. Apart from the fact they can be more easily harvested than mesenchymal stem cells (MSCs), hADSCs secrete nearly all of the growth factors that take part in normal wound healing [7,8,9,10]. After implantation, these cells may remain viable at the wound site and secrete growth factors in a continuous and regulated manner in response to environmental cues, just as it occurs in the natural wound healing process [11]. Consequently, at the injury site, implanted cells that undergo differentiation generate not only an inert filling tissue, but they are able to stimulate cell recruitment from stem cell niches in order to aesthetically restore the site of injury in a paracrine manner (by secretion of growth factors and cytokines). These observations suggest that hADSCs could be better candidates for tissue engineering applications than other traditional cell sources.

A great number of biomaterials have been used in the perspective of tissue reconstruction, but collagen-based scaffolds were proven to provide the best results [12]. Furthermore, the addition of bioactive molecules of natural origin in the composition of the currently used biomaterials could improve the biological performances of the resulting scaffold in terms of cellular adhesion, proliferation potential, extracellular matrix synthesis, intercellular signaling, modulation of stem cells differentiation, *etc*. In the context of these modern strategies, an attractive source of natural polymers with great physico-chemical properties is the silk isolated from *Bombyx mori* cocoons. These fibers are composed primarily of two types of proteins: fibroin, the core filaments of silk, and sericin, the antigenic gum-like protein surrounding the fibers [13]. Silk sericin (SS) is a granular protein with adhesive and gelatin-like characteristics, which was shown to be responsible for the proliferation and attachment of several mammalian cell lines [14,15,16], as well as for the activation of collagen production, both *in vitro* and *in vivo* [17,18,19].

Although early reports claimed that sericin was responsible for triggering an immune reaction, Nazarov *et al.* [20] showed that only the physical association of sericin and fibroin can produce an inflammatory response. Furthermore, it was reported that the SS peptides have no immunogenicity *in vivo* and can be used effectively in biomedical applications [21,22].

Since sericin was shown to have good hydrophilic properties, compatibility and biodegradation, Aramwit *et al.* [18,23] recommended its use as a wound healing agent. Low levels of sericin released from the scaffold proved to be beneficial, as sericin can activate collagen production in wounds. Sericin generates fragile materials that are not suitable for use in medical applications, but Mandal *et al.* [24] demonstrated that after blending with gelatin, the newly designed scaffolds are good candidates for tissue engineering applications. These scaffolds can easily be tuned by varying their compositions to obtain the desired level of sericin release, which may be significant in terms of wound healing and tissue engineering [24].

Collagen and sericin were associated for the first time in a 3D scaffold, which was characterized in terms of physico-chemical, morphological and mechanical properties by Lungu *et al.* [25]. The goal of our study was to evaluate the biocompatibility of this new designed superporous scaffold in terms of cell adhesion, proliferation potential and extracellular matrix production. In addition, we also aimed to investigate hADSCs’ adipogenic potential in contact with collagen based scaffolds in the presence and in the absence of sericin in the prospective use for ATE applications.

## 2. Results and Discussion

### 2.1. Biocompatibility Assessment of Coll and Coll-SS Biomatrices in Contact with hADSCs

In our experiments, we defined as bioconstructs the porous 3D hybrids resulting after collagen (Coll) and collagen-sericin (Coll-SS) biomaterials were put in contact with hADSCs. Although hADSCs were seeded on the surface of the biomatrices, cells were allowed to diffuse through the interconnected pores of the scaffolds and to distribute within the structures, resulting in 3D culture systems.

The biocompatibility of Coll-SS *versus* Coll was tested in terms of viability and proliferation, by double fluorescence Live/Dead staining and quantitative MTT assay. In addition, the cytotoxic potential of both matrices on hADSCs was evaluated using lactate dehydrogenase (LDH) spectrophotometric test.

#### 2.1.1. Qualitative Evaluation of hADSCs’ Viability and Proliferation Potential in Coll and Coll-SS

**Figure 1:**
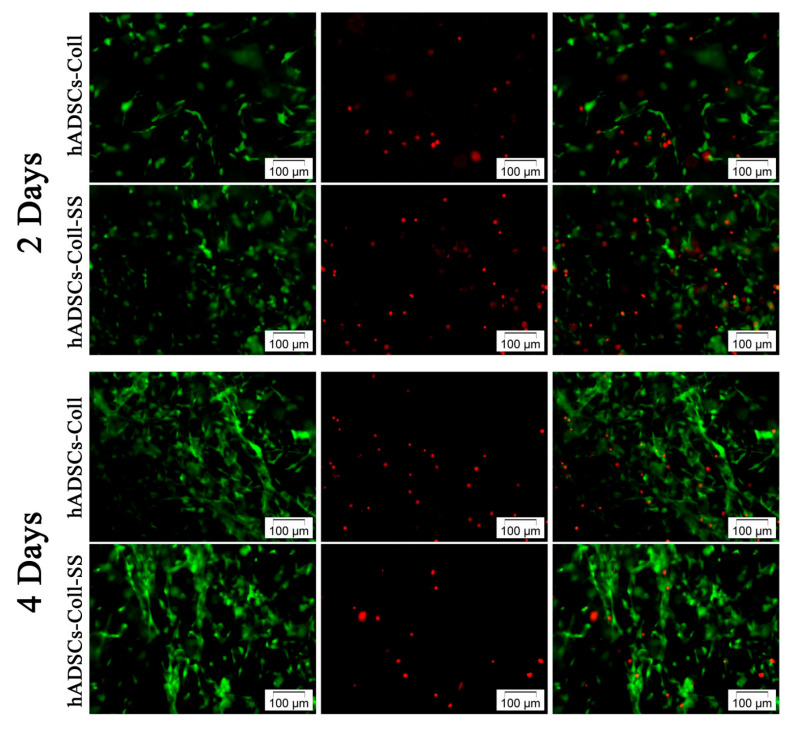

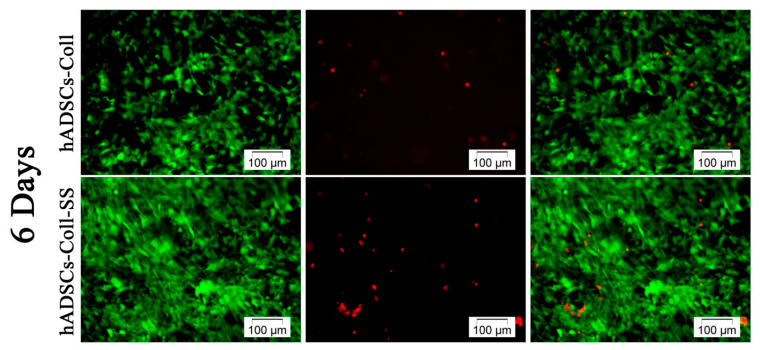
Fluorescence microscopy detection of live (green-labeled) and dead (red-labeled) human adipose-derived stem cells (hADSCs) in contact with Coll and Coll-SS biomatrices at 2, 4 and 6 days post seeding.

In order to examine cell survival up to one week, the viability of hADSCs in contact with Coll and Coll-SS 3D systems was evaluated at 2, 4 and 6 days post seeding using Live/Dead assay. As shown in Figure 1, the ratio between the viable (green labeled) and the dead (red labeled) cells was found to be constantly positive, whereas a higher cellular density was revealed on Coll-SS than on the control system. Consequently, hADSCs on the surface of Coll-SS reached a confluent monolayer faster than the cells on top of Coll scaffold, thus displaying a higher proliferative potential in the presence of sericin. In addition, in the context of these proliferative 3D cultures, the amount of dead cells observed was lower at 6 days, as compared to 2 and 4 days post-seeding in both bioconstructs, suggesting that hADSCs were able to adapt to the 3D microenvironment provided by the scaffolds. This fluorescence microscopy investigation also revealed the fibroblast-like morphology of the green-labeled living cells. However, cell density on Coll-SS was higher than on Coll system at 2 days post-seeding, probably due to the sticky properties of sericin. These observations are in accordance with previous findings [15,19], which stated that sericin enhances cell proliferation and attachment.

#### 2.1.2. Quantitative Evaluation of hADSCs’ Viability and Proliferation Potential in Coll and Coll-SS

In order to confirm the viability and proliferation results revealed by Live/Dead assay, a MTT test was employed as a more accurate approach. The spectrophotometric determination of the formazan concentrations was issued for up to one week, at 2, 4 and 6 days post seeding.

**Figure 2:**
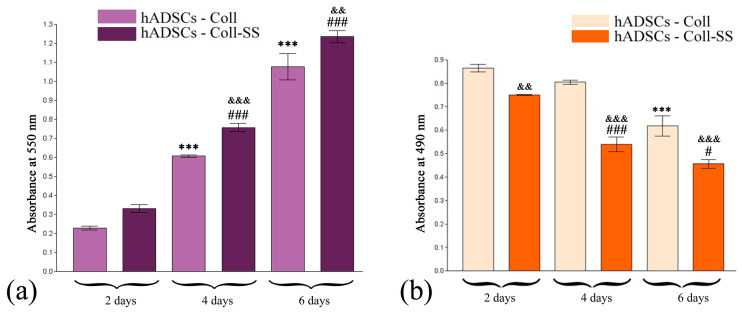
The quantification of (**a**) hADSCs proliferation rate in Coll and Coll-SS biomatrices as revealed by MTT test. *** *p* < 0.001 (Coll bioconstruct: 4 days *vs.* 2 days and 6 days *vs.* 4 days); ### *p* < 0.001 (Coll-SS bioconstruct: 4 days *vs.* 2 days and 6 days *vs.* 4 days); && *p* < 0.01 (Coll-SS bioconstruct *vs.* Coll bioconstruct: 6 days); &&& *p* < 0.001 (Coll-SS bioconstruct *vs.* Coll bioconstruct: 4 days)]; (**b**) the cytotoxic potential of Coll and Coll-SS biomatrices on hADSCs as revealed by LDH assay. *** *p* < 0.001 (Coll bioconstruct: 6 days *vs.* 4 days); # *p* < 0.05 (Coll-SS bioconstruct: 6 days *vs.* 4 days); ### *p* < 0.001(Coll-SS bioconstruct: 4 days *vs.* 2 days); && *p* < 0.01 (Coll-SS bioconstruct *vs.* Coll bioconstruct: 2 days); &&& *p* < 0.001 (Coll-SS bioconstruct *vs.* Coll bioconstruct: 4 days and 6 days).

Although the viability of hADSCs in contact with Coll-SS was found to be permanently increased in a time-dependent manner as compared to Coll matrix (control), statistically significant differences were found only at 4 (*p* < 0.001) and at 6 (*p* < 0.01) days of culture. As shown in Figure 2a, the proliferation of cells seeded in Coll-SS registered a significant increase at 4 days as compared to 2 days (*p* < 0.001) and at 6 days as compared to 4 days (*p* < 0.001). The same profile, but with lower values, was obtained for cells in contact with the control scaffold Coll.

#### 2.1.3. Cytotoxic Potential of Coll and Coll-SS on hADSCs

Based on the evaluation of Coll and Coll-SS cytotoxic potential on hADSCs up to one week, the activity of LDH in the culture media was found to be increased at 2 days post seeding as compared to 4 and 6 days in both bioconstructs. The statistical differences between Coll and Coll-SS were lower at 2 days of culture (*p* < 0.01), as compared to 4 days (*p* < 0.001) and 6 days (*p* < 0.001) of culture. Further on, LDH activity in Coll-SS system decreased dramatically in the first 4 days of culture (*p* < 0.001) and maintained this descending profile up to 6 days, but at a lower rate (*p* < 0.05). Additionally, the cytotoxic effect of Coll showed an overall decreasing trend, displaying only one significant difference between 4 and 6 days of culture (*p* < 0.001). These findings suggest that the collagen-based biomaterials displayed a lower cytotoxic effect on hADSCs when sericin was added in their composition.

The viability and proliferation data together with the quantification of the cytotoxic potential may suggest that hADSCs require a short period of time to accommodate to the new 3D microenvironment provided by both Coll and Coll-SS.

### 2.2. Assessment of hADSCs Adipogenic Differentiation Potential in Coll and Coll-SS Biomatrices

hADSCs-Coll and hADSCs-Coll-SS bioconstructs were subjected to adipogenesis and consequently investigated in terms of 3D system morphology, neutral lipids accumulation and gene, as well as protein expression of adipogenic specific markers at 3, 7, 14, 21 and 28 days post-induction.

#### 2.2.1. Morphological Evaluation of hADSCs-Scaffold Bioconstructs during Adipogenesis

Based on a previous study, Lungu *et al.* [25] reported that the sericin content strongly influenced the structure-properties relationship of Coll-SS scaffolds and confirmed their superporous nature with pore size between 60 and 130 μm for Coll and 50 to 90 μm for Coll-SS scaffolds. Moreover, the swelling properties, together with the particular architectural characteristics of Coll-SS, influence the *in vitro* degradation and thermal stability of these materials [25].

Although hADSCs were seeded on the surface of Coll and Coll-SS scaffolds, scanning electron microscopy (SEM) analysis performed on the transversal sections of these bioconstructs at 7, 14 and 21 days post induction revealed that cells populated the entire structure. The cell distribution inside the biomatrices could be related to the porous structure, particularly to the interconnectivity of the pores, as previously shown [25].

The behavior of the biomaterials in the adipogenic conditions used by us was revealed through SEM (data not shown). Therefore, Coll biomatrix showed a slow and constant degradation rate, whereas Coll-SS strongly compacted, probably due to the release of sericin from the original scaffold into the medium. Considering wound healing applications, low levels of sericin released from the scaffold could be beneficial, as sericin is able to promote collagen production in wounds [19,23], but, at the same time, the matrix should also maintain its stability [26]. What is more, Mandal *et al.* [24] reported that scaffolds having sericin in their composition could easily be adjusted in order to obtain the desired level of sericin release. These features may be significant in terms of wound healing and tissue engineering [23].

#### 2.2.2. Evaluation of the Intracellular Lipid Droplet Accumulation

**Figure 3:**
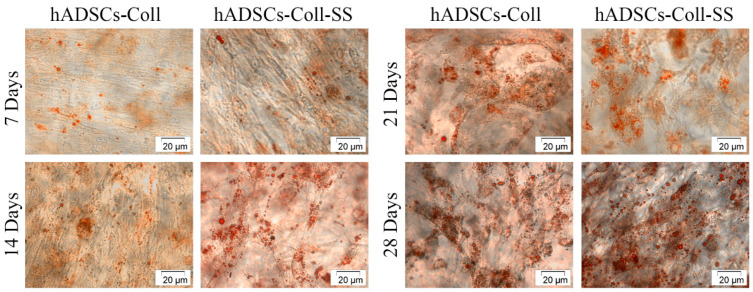
Contrast-phase micrographs of Oil Red O stained hADSCs-Coll and hADSCs-Coll-SS bioconstructs after 7, 14, 21 and 28 days post adipogenic induction.

Contrast-phase microscopy images of Oil Red O stained bioconstructs revealed that the neutral lipid accumulation started at 7 days post adipogenic induction in cells loaded in both Coll and Coll-SS scaffolds. As shown in Figure 3, the number of lipid droplets, as well as their volume, increased during the adipogenic process in both bioconstructs, thus confirming the SEM observations regarding cellular morphology.

No significant differences were observed in terms of intracytoplasmatic lipid droplets accumulation in cells undergoing adipogenesis in Coll and Coll-SS up to 28 days.

#### 2.2.3. PPARγ2, FAS, aP2 and Perilipin Gene Expression Profiles

At the molecular level, adipogenesis is regulated by a complex transcriptional cascade. Peroxisome proliferator-activated receptor γ2 (PPARγ2), the key inducer of the adipogenic differentiation process, promotes terminal differentiation by activating the transcription of the battery of genes involved in inducing and maintaining the adipocyte phenotype [27]. These downstream targets include fatty acid synthase (FAS), adipocyte fatty acid–binding protein (aP2), perilipin, lipoprotein lipase (LPL), fatty acid transport protein-1 (FATP-1), the adipocytokines (adiponectin, leptin, resistin) [28,29], *etc*.

In order to evaluate the evolution of the process in our adipogenic conditions, the expression pattern of early and late adipogenic markers was investigated up to 28 days using the RealTime RT-PCR technique.

**Figure 4:**
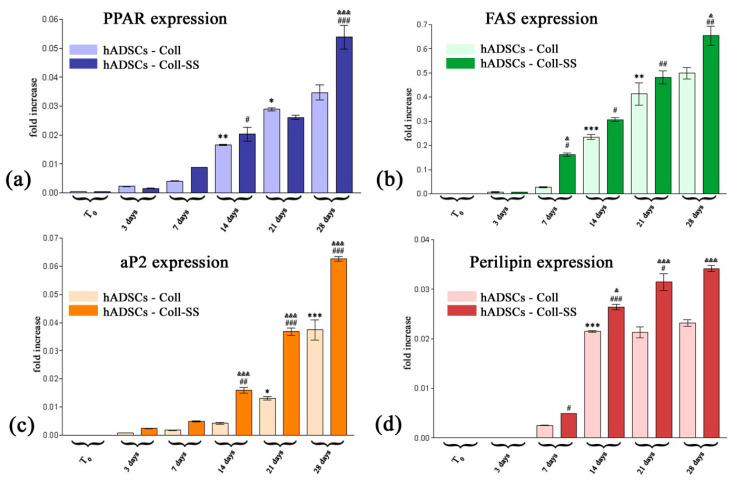
Gene expression profiles of (**a**) PPARγ2 (* *p* < 0.05 (Coll bioconstruct: 21 days *vs.* 14 days); ** *p* < 0.01 (Coll bioconstruct: 14 days *vs.* 7 days); # *p* < 0.05 (Coll-SS bioconstruct: 14 days *vs.* 7 days); ### *p* < 0.001 (Coll-SS bioconstruct: 28 days *vs.* 21 days); &&& *p* < 0.001 (Coll-SS bioconstruct *vs.* Coll bioconstruct: 28 days)). (**b**) FAS (** *p* < 0.01 (Coll bioconstruct: 21 days *vs.* 14 days); *** *p* < 0.001 (Coll bioconstruct: 14 days *vs.* 7 days); # *p* < 0.05 (Coll-SS bioconstruct: 7 days *vs.* 3 days and 14 days *vs.* 7 days); ## *p* < 0.01 (Coll-SS bioconstruct: 21 days *vs.* 14 days and 28 days *vs.* 21 days); & *p* < 0.05 (Coll-SS bioconstruct *vs.* Coll bioconstruct: 7 days and 28 days)). (**c**) aP2 (* *p* < 0.05 (Coll bioconstruct: 21 days *vs.* 14 days); *** *p* < 0.001 (Coll bioconstruct: 28 days *vs.* 21 days); ## *p* < 0.01 (Coll-SS bioconstruct: 14 days *vs.* 7 days); ### *p* < 0.001 (Coll-SS bioconstruct: 21 days *vs.* 14 days and 28 days *vs.* 21 days); &&& *p* < 0.001 (Coll-SS bioconstruct *vs.* Coll bioconstruct: 14 days, 21 days and 28 days)). And (**d**) perilipin (*** *p* < 0.001 (Coll bioconstruct: 14 days *vs.* 7 days); # *p* < 0.05 (Coll-SS bioconstruct: 7 days *vs.* 3 days and 21 days *vs.* 14 days); ### *p* < 0.001 (Coll-SS bioconstruct: 14 days *vs.* 7 days); & *p* < 0.05 (Coll-SS bioconstruct *vs.* Coll bioconstruct: 14 days); &&& *p* < 0.001 (Coll-SS bioconstruct *vs.* Coll bioconstruct: 21 days and 28 days)) as quantified by RealTime RT-PCR.

In our study, *PPARγ2* transcript levels were detected, including in the samples harvested before inducing *in vitro* adipogenesis in both hADSCs-Coll and hADSCs-Coll-SS bioconstructs. This feature suggests that *PPARγ2* gene is active at basal levels independent of the presence of pro-adipogenic conditions and confirms its potential as master activator and regulator of adipogenesis. Although our results show that PPARγ2 expression describes an ascendant trend post induction (Figure 4a), statistical significant increases in gene expression were registered at 14 days, both for Coll (*p* < 0.01) and for Coll-SS (*p* < 0.05) systems, when compared to 7 days. Furthermore, we detected a significant upregulated PPARγ2 profile (*p* < 0.001) in the presence of sericin (hADSCs-Coll-SS bioconstruct) at 28 days, as compared to 21 days. This is in contrast with PPARγ2 mRNA levels obtained for control (hADSCs-Coll bioconstruct), since an increase (*p* < 0.05) was detected at 21 days *versus* 14 days, but no other significant upregulation was noticed until the end of the experiment. When comparing PPARγ2 expression pattern in the presence (hADSCs-Coll-SS bioconstruct) or absence of sericin (hADSCs-Coll construct), the statistical important differences (*p* < 0.001) occurred at 28 days post adipogenic induction. This gene expression profile is in accordance with the PPARγ2 expression pattern we previously reported in a 2D culture system [30]. However, a slower activation of PPARγ2 gene was noticed in 3D collagen-based bioconstructs than in 2D systems, probably due to the scaffold’s impediments and lower diffusion rates of adipogenic inducers towards the cells. Consequently, a modulation of the adipogenic conditions usually used in 2D culture systems is required when targeting 3D hADSCs-scaffold differentiation in the context of ATE, as we previously described [30].

Once activated, PPARγ2 induced the transcription of FAS, aP2 and perilipin, which act together in order to synthesize, transport and mediate triacylglycerol (TAG) metabolism, respectively. Thus, we first detected the activation of FAS gene, one of the downstream targets of PPARγ2 [29], at 7 days post-induction in both bioconstructs (Figure 4b), but to a higher extent (*p* < 0.05) in the presence of sericin, as compared to the control system. Moreover, at 7 days of adipogenic induction, a significant change in FAS gene expression was reported only in hADSCs-Coll-SS bioconstruct as compared to 3 days post-induction (*p* < 0.05). For this bioconstruct, a further significant increase was registered between 7 and 14 days (*p* < 0.05), while for the hADSCs-Coll system, the first statistically significant increase was detected later, at 14 days, as compared to the previous time point (*p* < 0.001). This upregulated profile registered a constant and statistically significant increase (*p* < 0.01) in both bioconstructs, during 14–21 days interval, suggesting the constant requirement of free fatty acids synthesis throughout the adipogenic differentiation process, independent of sericin influence. However, the FAS mRNA levels continued to increase up to 28 days post-induction in the presence of sericin (*p* < 0.01), while the transcript levels corresponding to the control sample at 28 days were comparable to those at 21 days of adipogenesis. This difference registered between FAS transcript expression at 28 days in the presence and absence of sericin proved to have statistical significance (*p* < 0.05), highlighting the possible influence of sericin on TAG synthesis during *in vitro* adipogenesis.

Fatty acid binding protein aP2, required for the transport of TAG across internal membranes, was detected at low levels starting with day 7 post-adipogenic induction (Figure 4c). However, the first significant differences (*p* < 0.01) in aP2 gene expression were revealed at 14 days in samples harvested from hADSCs-Coll-SS bioconstruct, as compared to 7 days. This upregulated pattern of expression was maintained for both 14–21days (*p* < 0.001) and 21–28 days (*p* < 0.001) time intervals in the presence of SS, while the cells in Coll scaffolds expressed lower levels of aP2 and with lower statistical significances in 14–21 days (*p* < 0.05) and 21–28 days (*p* < 0.001) intervals. Overall, important statistical differences (*p* < 0.001) in aP2 transcript levels were noticed between hADSCs-Coll-SS and hADSCs-Coll bioconstructs at 14, 21 and 28 days after adipogenic induction in our culture conditions, thus confirming that aP2 is a late adipogenic marker and raising the hypothesis that sericin is able to influence its expression in 3D culture systems.

The lipid droplet associated protein perilipin was first statistically detected in Coll-SS system at 7 days post adipogenic induction (*p* < 0.05), as compared to 3 days, whereas the cells loaded in Coll scaffold significantly expressed perilipin later, at 14 days (*p* < 0.001), compared to 7 days (Figure 4d). Consecutively to this activation, perilipin expression profile corresponding to the control Coll system remained approximately constant up to 28 days, whereas perilipin transcript levels in Coll-SS statistically increased between 7 and 14 days (*p* < 0.001) and between 14 and 21 days (*p* < 0.05) of adipogenesis. Overall, our results suggest that the expression pattern of perilipin is highly influenced by the presence of sericin in 3D systems undergoing adipogenesis, since the most significant statistical differences appeared between the samples recovered simultaneously from Coll and Coll-SS biomatrices at 14 (*p* < 0.05), 21 (*p* < 0.001) and 28 (*p* < 0.001) days post adipogenic induction.

All these data show that the addition of sericin in the composition of implantable collagen-based biomatrices enhances the activation of PPARγ2, which triggers the activation of FAS, aP2 and perilipin adipogenic markers. This results in a higher efficiency rate of adipogenesis in our conditions, as compared to a pure collagen system.

#### 2.2.4. Late Adipogenic Marker Perilipin Protein Expression during Adipogenesis in hADSCs-Coll and hADSCs-Coll-SS Bioconstructs

Perilipin protein expression was qualitatively analyzed by fluorescence and confocal microscopy and quantitatively evaluated by flow cytometric detection.

**Figure 5:**
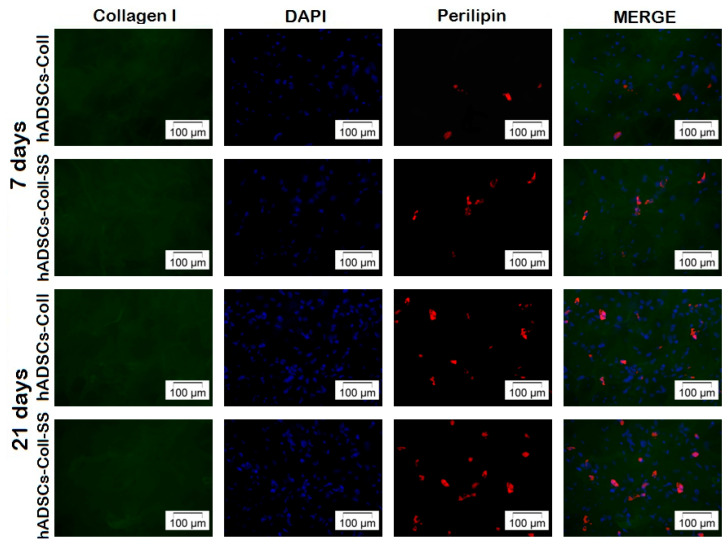
Fluorescence microscopy micrographs revealing perilipin expression after 7 and 21 days of adipogenesis in both hADSCs-Coll and hADSCs-Coll-SS constructs.

Fluorescence microscopy analysis (Figure 5), performed after 7 and 21 days of *in vitro* adipogenic induction, revealed gradual accumulation of perilipin during the process and perilipin cellular distribution. After 7 days of *in vitro* adipogenic differentiation, a very low amount of perilipin was found in both constructs, whereas a higher amount of perilipin was detected in the presence of sericin (hADSCs-Coll-SS construct) than in the pure collagen construct after 21 days of induction. Additionally, adipogenic differentiated cells distribution in the context of Coll and Coll-SS 3D scaffolds was possible due to the immunostaining of the collagen found in the composition of both matrices, simultaneously with perilipin labeling.

**Figure 6:**
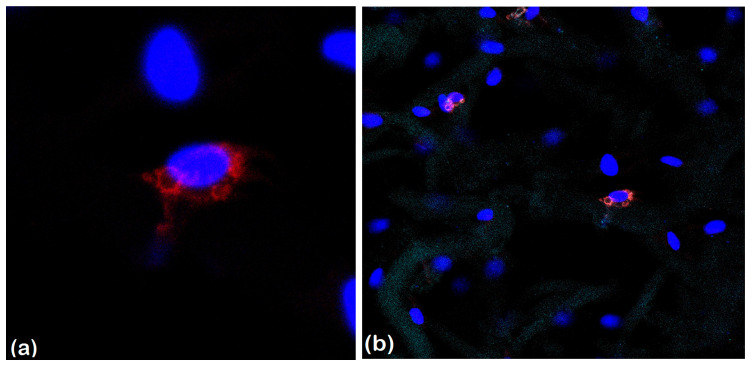
Confocal images revealing (**a**) perilipin distribution around the nucleus in a hADSC-Coll-SS construct exposed to adipogenic differentiation *in vitro* for 14 days and (**b**) hADSCs-Coll-SS construct after 14 days of adipogenesis triple stained for collagen type I (green), perilipin (red) and nuclei (blue).

Furthermore, confocal microscopy revealed perilipin distribution at the periphery of the lipid droplets formed in maturating adipocytes and also the tridimensional nature of the adipogenic process inside hADSCs-Coll-SS construct (Figure 6).

**Figure 7:**
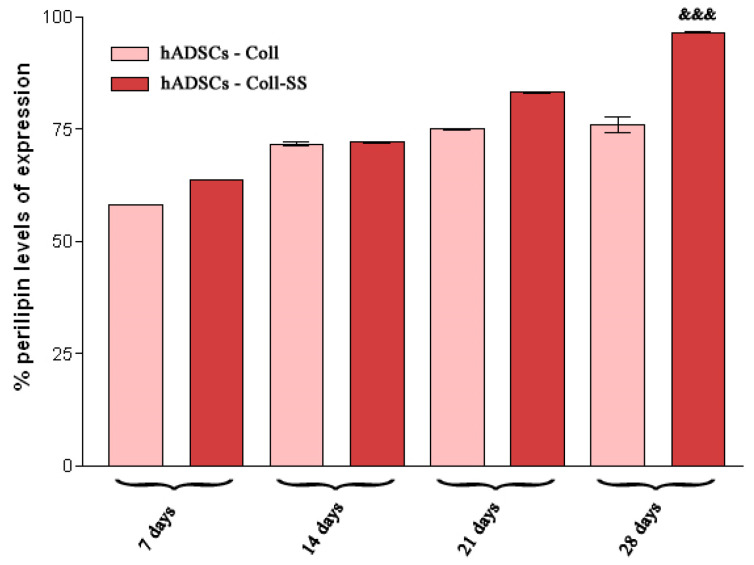
Perilipin expression in cells undergoing adipogenesis for up to 28 days in Coll *vs.* Coll-SS biomatrices, as revealed by flow cytometry [&&& *p* < 0.001 (Coll-SS bioconstruct *vs.* Coll bioconstruct: 28 days)].

Perilipin expression in hADSCs-Coll and hADSCs-Coll-SS was studied by flow cytometry after harvesting cells at 3, 7, 14, 21 and 28 days post-adipogenic induction. The results (Figure 7) showed that perilipin was first detected at 7 days of adipogenesis in both 3D culture systems, confirming the observations regarding the lipid droplets accumulation revealed by Oil Red O staining. Notably, perilipin levels of expression registered a gradually increase up to 28 days in Coll-SS bioconstruct, whereas the levels were constant between 14 and 28 days in the control biomatrix.

Overall, perilipin expression was found to be higher for cells that differentiated in the presence of sericin (Col-SS biomatrix) than for those undergoing adipogenesis in pure collagen bioconstructs (Coll biomatrix), with a statistical significance at 28 days post induction (*p* < 0.001).

Regarding the importance of using hADSCs-Coll-SS constructs in soft tissue reconstruction applications rather than autologous fat implants, there are certain aspects that need to be emphasized. In the context of wound healing and dermal reconstruction [31], common treatment approaches for soft tissue reconstruction include the implantation of autografts, allografts and xenografts. The low number of donors, antigenicity and donor site morbidity limit the use of these soft tissue substitutes. Concerning autologous fat implantation particularly, which is considered to be the ideal filling material in terms of biocompatibility [2], a serious limitation is the amount of fat needed to fill tissue defects or to cover deep and extended lesions. If high amounts of fat tissue must be harvested from a patient for an autologous implant, a low amount of fat tissue is required for obtaining sufficient hADSCs in order to preseed a collagen-based biomatrix, such as Coll-SS, before *in vivo* implantation. This is due to the fact that cells have the ability to proliferate, under sericin’s pro-proliferative effect, as already shown [19]. What is more, another limitation is the unpredictability of fat tissue survival-graft resorption over time, and the lack of sufficient revascularization [3,4] may cause soft tissue reconstruction failure.

In contrast, the use of collagen-based biomatrices preseeded with hADSCs would greatly improve the quality of *in vivo* soft tissue reconstruction. To begin with, the biomatrix would ensure a mechanical and structural support to cells, opposed to the autologous fat implants, which can be reabsorbed, causing the reconstruction site to deform. In general, the artificial 3D scaffold provides a structure on which seeded cells can organize and develop into the desired tissue for implantation. Once implanted in the soft tissue defect, the biodegradable scaffold provides an initial biomechanical structure for the replacement tissue until the cells produce their own extracellular matrix. During the deposition, organization and formation of the newly generated extracellular matrix, the scaffold is either degraded or metabolized [32].

Apart from ensuring a support for cells *in vivo*, an implanted collagen-sericin matrix could potentially stimulate the natural local collagen secretion [17,18] and the specific components of adipose tissue matrix, thus displaying pro-adipogenic properties. Additionally, silk sericin is biocompatible, biodegradable and has good hydrophilic properties, thus it can be used as a wound healing agent [18]. Approximately 30% of the amino acid content of sericin is serine, a key moistening factor [33]. Moist environments accelerate the dynamic process of rapid wound healing [34]. Besides a natural moisturizing agent, sericin also possesses antibacterial, antioxidant, anticoagulating and antiwrinkle activity and enhances the proliferation of mammalian cells [31].

What is more, Coll-SS biomatrix is designed as a combination of two natural compounds in a ratio that allows biodegradability rate control in such a manner that ensures enough time for novel *in situ* tissue formation. This is in accordance with the results obtained by Mandal *et al.* [24]. Furthermore, cells are homogeneously distributed when being dispersed in a 3D matrix. Thus, it is expected that growth factors, differentiation factors or key molecules from the *in vivo* environment can reach cells more easily than in an autologous fat implant.

Finally, in our opinion, the most important challenge in current ATE is to aesthetically restore the soft tissue lesion site and to functionally reconstruct all tissue layers, including the dermal layer, not only to fill the missing tissue with autologous fat. Assuming hADSCs capacity to recruit other cells in a paracrine manner [35], local regeneration of all cellular types required in order to achieve a functional tissue would be possible. Thus, at this moment, tissue reconstruction using collagen-based biomatrices preseeded with hADSCs appears to be more efficient than autologous fat implantation.

## 3. Experimental Section

### 3.1. Cell Culture Model

hADSCs were isolated from subcutaneous adipose tissue by digestion with 0.01% collagenase type I solution [36] and cultured in Dulbecco’s Modified Eagle’s Medium (DMEM) (Sigma-Aldrich Co., Steinheim, Germany) supplemented with 10% fetal bovine serum (FBS). The heterogenous primary population was purified during subcultivation by several passages based on the adherence properties hADSCs have in contrast with hematopoietic precursor cells, resulting in a hADSCs enriched culture starting with the 3rd passage [30,37]. All further tests were performed using cells in 3–7 passages.

The human abdominal white adipose tissue was obtained from 3 female patients with ages between 35 and 38 years undergoing elective liposuction, and all the medical procedures were performed in compliance with the Helsinki Declaration, with the approval of the Emergency Hospital for Plastic Surgery and Burns Ethical Committee (reference No. 3076/10.06.2010). All subjects were in good health and provided written consent before participation in the study.

### 3.2. Preparation of 3D hADSC Cultures within Coll and Coll-SS Biomatrices

Type I fibrillar collagen was extracted from calf hide as a gel with an initial concentration of 1.54% (*w*/*w*) by acid and alkaline treatments, as previously described [38]. Sericin silkworm was purchased from Sigma-Aldrich (Shinagawa-Ku, Tokyo, Japan). Glutaraldehyde (GA) was received from Merck (Darmstadt, Germany).

Superporous biomatrices based on collagen and sericin were prepared, as previously described by Lungu *et al.* [25]. Briefly, 40% Silk sericin (SS) (reported to collagen dry substance) was added to collagen gel, keeping the collagen concentration constant (1.2%). The pH was maintained at 7.4 during sample preparations. The gels were cross-linked with 0.5% GA (reported to the weight of dry collagen), then cast in disposable polystyrene dishes and kept at 4 °C for 24 h. The obtained hydrogels were freeze-dried and biomatrices (Coll as a reference sample and Coll-SS with 40% SS reported to collagen) with an initial surface of 3.1 cm^2^ and 4 mm in thickness were obtained.

The novel characterized Coll-SS biomatrix was further used in our studies and was permanently compared in terms of biocompatibility and adipogenic potential to a pure collagen matrix, which served as control.

hADSCs in the 3rd passage were seeded on top of Coll and Coll-SS biomatrices at an initial density of 1.4 × 10^5^ cells/cm^2^. The cell suspension was allowed to diffuse through the scaffolds in order for the cells to adhere to the biomaterial. After 1 h, the resulting 3D bioconstructs were incubated in standard conditions of cultivation in DMEM supplemented with 10% FBS.

### 3.3. Biocompatibility Assessment

The viability and proliferation potential of hADSCs in contact with Coll and Coll-SS scaffolds were assessed using qualitative Live/Dead Assay and a quantitative MTT test. The cytotoxic potential of the biomaterials on hADSCs was evaluated by spectrophotometric quantification of the LDH activity in culture medium.

#### 3.3.1. Live/Dead Fluorescence Microscopy Assay

Cell viability and proliferation within the 3D culture systems was evaluated by fluorescence microscopy using Live/Dead Kit (Invitrogen, Life Technologies, Foster City, CA, USA). This method allows the simultaneous detection of both live and dead cells with calcein acetoxymethyl (calcein AM) and ethidium bromide dyes provided in the kit. Calcein AM is a non-fluorescent and permeable reagent, which is converted by the intracellular esterases to the intensely green fluorescent calcein (ex/em: ~495 nm/~515 nm). Ethidium bromide enters the cells with damaged membrane, producing a bright red fluorescence when binding to nucleic acids (ex/em: ~495 nm/~635 nm).

Briefly, at 2, 4 and 6 days post seeding, the hADSCs-Coll and hADSCs-Coll-SS bioconstructs were incubated with a staining solution prepared according to the manufacturer’s instructions for 15 min. Next, the stained 3D cultures were analyzed by fluorescence microscopy using an Olympus IX71 inverted microscope, and images were captured with Cell F Imaging Software (Olympus: Hamburg, Germany, 2008).

#### 3.3.2. MTT Spectrophotometric Test

The viability and the proliferation capacity of the cells within the biomatrices were quantitatively assessed by MTT assay at 2, 4 and 6 days post seeding, in order to validate the findings revealed by Live/Dead assay. This test is based on the reduction of a tetrazolium salt solution—MTT—to purple formazan by metabolically active cells. Both hADSC-Coll and hADSC-Coll-SS bioconstructs were incubated for 24 h in 1 mg/mL MTT solution (Sigma Aldrich Co., Steinheim, Germany). The concentration of the formazan produced by the metabolically active cells was spectrophotometrically quantified at 550 nm (Appliskan Thermo Scientific, Waltham, MA, USA), after solubilization in isopropanol. The result was a sensitive assay with a colorimetric signal proportional to the viable cell number.

#### 3.3.3. LDH Spectrophotometric Assay

The cytotoxic potential of Coll and Coll-SS on hADSCs was evaluated using “*In vitro* toxicology assay kit lactate dehydrogenase based” (Sigma Aldrich Co., Steinheim, Germany), considering the spectrophotometric detection of lactate dehydrogenase (LDH), which is released in the culture medium by the cells with damaged membranes. As LDH is a cytosolic enzyme, its detection in the culture media is correlated with membrane damage and the potential cytotoxicity of the cellular environment.

At 2, 4 and 6 days post seeding, the culture media were harvested and mixed with the solutions provided in the kit, following instructions. After 20 min of incubation, the reaction was stopped with 1N hydrochloric acid (HCl) and the LDH enzymatic activity was determined by measuring the optic density of the resulting solution at 490 nm (Appliskan Thermo Scientific).

### 3.4. hADSCs Adipogenic Potential in Coll and Coll-SS Biomatrices

The 3rd passage hADSCs were analyzed for their capacity to differentiate towards the adipogenic lineage when embedded in Coll and Coll-SS biomatrices. Therefore, the resulted bioconstructs were exposed for up to 28 days to an optimized adipogenic protocol [30], designed to modulate the kinetics of the differentiation process. This protocol is based on the administration of the main inducers (isobutyl methyl xanthine [IBMX], dexamethasone [DEX], troglitazone) separately from the pro-adipogenic supporting molecules (biotin, indomethacine, hydrocortisone, triiodothyronine and transferrin). In this study, adipogenesis was evaluated in terms of 3D bioconstruct morphology, intracellular lipid accumulation and adipogenic markers gene and protein expression.

#### 3.4.1. SEM of Collagen-Based Scaffolds Populated with hADSCs during Adipogenesis

The 3D constructs were subjected to adipogenesis for 7, 14 and 21 days and fixed for 6 h at 4 °C with 2.5% glutaraldehyde (Sigma-Aldrich, Co., Steinheim, Germany) in PBS. After rinsing with double distilled water, the samples were dried at 20 °C and 0.1 mbar pressure for 4 h in a 24-LSC Martin Christ laboratory freeze dryer. Then, the samples were coated with gold and imaged using a FEI Quanta Inspect F with field emission gun (FEG), operating in SEM mode. The microscope was driven with an acceleration voltage of 30 kV and a working distance of 10 mm detecting secondary electrons.

#### 3.4.2. Oil Red O Staining for Lipid Droplet Accumulation

The accumulation of cytoplasmic droplets of neutral lipids was assessed by Oil Red O staining at 7, 14, 21 and 28 days post-induction. Briefly, hADSCs-Coll and hADSCs-Coll-SS were fixed for 8 h with 4% para-formaldehyde (PFA). After permeabilization with 2% bovine serum albumin (BSA)/0.1% Triton X-100 solution, both bioconstructs were incubated with Oil Red O solution (5 mg/ml in 60% isopropanol, diluted 3:2 with tap water) for 24 h at 4 °C. The assessment of lipid droplets accumulation was revealed by phase contrast microscopy (Olympus IX71) and Cell F Imaging Software (Olympus).

#### 3.4.3. RealTime Quantification for PPARγ2, FAS, aP2 and Perilipin Adipogenic Markers

hADSCs were harvested from Coll and Coll-SS scaffolds by digestion with 0.01% collagenase type I solution at T0 (zero time when adipogenic induction cocktail was added to the culture), 3, 7, 14, 21 and 28 days. After centrifugation, total RNA was isolated using RNA PureLink Mini Kit (Ambion, Life Technologies, Foster City, CA, USA), according to the manufacturer’s protocols, and tested for integrity on BioAnalyzer 2100 (Agilent Technologies, Waldbronn, Germany) and purity on NanoDrop spectrophotometer (Shimadzu, Duisburg, Germany). One microgramme of total cellular RNA was reverse transcribed to corresponding cDNA using iScript cDNA Synthesis kit (BioRad, Hercules, CA, USA). The sequences of our target gene primers are presented in Table 1, and the melting temperature was optimized on a Corbett thermocycler, in 52–62 °C range of temperatures, based on standard PCR components (GoTaq DNA Polymerase kit) provided by Promega, Madison, WI. The gradient PCR amplicons were subjected to 2% agarose electrophoresis, followed by ethidium bromide (Roth, Karlsruhe, Germany) staining.

The PPARγ2, FAS, aP2 and perilipin expression patterns during adipogenesis were revealed up to 28 days post-induction by RealTime RT-PCR on a LightCycler 2.0 carrousel-based system using LightCycler Fast Start DNA Master SYBR Green I Kit (Roche, Mannheim, Germany). The adipogenic markers levels of expression were normalized to the glyceraldehyde 3-phosphate dehydrogenase (GAPDH) reference gene and permanently compared to mRNA levels of the same markers from normal adipose tissue.

#### 3.4.4. Qualitative and Quantitative Detection of Perilipin Late Adipogenic Marker

Perilipin expression during hADSCs adipogenic differentiation in both Coll and CollSS scaffolds was visualized by fluorescence microscopy at 7 and 21 days post induction. In order to fluorescently label the cells and the collagen-based scaffolds, both hADSCs-Coll and hADSCs-Coll-SS constructs were fixed with 4% PFA for 8 h and permeabilized with 2% BSA/0.1% Triton X-100 solution at 4 °C. Next, the constructs were incubated overnight with a mix formed of a rabbit polyclonal anti-perilipin antibody solution (SC-67164, 1:50, Santa-Cruz Biotechnology, Heidelberg, Germany) and a goat polyclonal anti-collagen I antibody solution (SC-25974, 1:500, Santa-Cruz Biotechnology, Heidelberg, Germany). Finally, the bioconstructs were exposed to secondary antibodies solutions for 30 min (TRITC conjugated goat anti rabbit, 1:50 and FITC conjugated mouse anti goat, 1:50, Santa-Cruz Biotechnology, Heidelberg, Germany). After cell nuclei were stained using DAPI for 5 min, the resulting labeled constructs were visualized in fluorescence microscopy using an Olympus IX71 inverted microscope, and images were captured with Cell F Imaging Software (Olympus).

The triple staining of hADSC-Coll-SS bioconstruct was also evaluated by confocal microscopy at 14 days post-adipogenic induction. Confocal imaging was performed using the Leica TCS-SP5 confocal scanner with 4 lasers and Leica Confocal Software offline.

For flow cytometric detection of perilipin at 7, 14, 21 and 28 days after adipogenic induction, 3D hADSCs-Coll and hADSCs-Coll-SS bioconstructs were first digested according to the protocol described above. After 10 min of centrifugation at 265× g, 1.4 × 10^5^ cells were fixed with 4% PFA and permeabilized with 2% BSA/0.1% Triton X-100 solution. Further on, the cells were incubated overnight with rabbit polyclonal anti-perilipin antibody solution (1:200, Santa-Cruz Biotechnology, Heidelberg, Germany), and the next day, they were stained with FITC conjugated goat anti-rabbit IgG1 secondary antibody (1:50, Santa-Cruz Biotechnology, Heidelberg, Germany) for 30 min. A mean value of 10,000 events was acquired on a FC500 Beckman Coulter cytometer and analyzed with CXP 2.2 software (Beckman Coulter, London, UK, 2009). In order to keep the same parameters throughout the entire experiment, the cytometer was calibrated with Flow Check fluorescent beads (Beckman Coulter) before each determination.

### 3.5. Statistical Analysis

The statistical evaluation of the data was done using the one-way ANOVA method followed by Bonferroni’s multiple comparison test. All experiments were performed in triplicate, and the results were expressed as a mean ± standard deviation (SD) using GraphPad Prism Software (version 3.03; GraphPad Software Inc., San Diego, CA, USA, 2002) for Windows. For the biocompatibility assays, *n* = 2, as all tests were performed on 2 sets of Coll-SS and Coll scaffolds, each one in triplicate, and for the differentiation studies, *n* = 3, as they were performed on 3 different sets of Coll-SS and Coll scaffolds, each time in triplicate. Differences between samples were considered statistically significant for *p* < 0.05 and highly significant for *p* < 0.001.

## 4. Conclusions

Modern ATE strategies consist in the design of implantable 3D cell-scaffold bioconstructs that allow *de novo* tissue formation and also meet all the criteria for wound healing therapies. In this study, we describe the biological performances of a novel porous biomatrix based on two natural compounds: collagen and sericin. For the first time, a spongious 60% collagen and 40% sericin scaffold and its pure collagen control were preseeded with hADSCs, and the resulting bioconstructs were subjected to biocompatibility and adipogenic potential investigations *in vitro*. The tested biomaterials were first populated with hADSCs by the diffusion of the cell suspension through the interconnected pores of the 3D structures, followed by their adhesion to the substrate. Our data showed that the addition of the sticky protein sericin enhanced the proliferation rate of the seeded cells, thus improving the biocompatibility of the Coll-SS scaffold. Furthermore, this study brought new valuable information on the *in vitro* adipogenic differentiation conducted in collagen-based biomatrices, in the presence or absence of sericin and the influence of the scaffold on the evolution of the process. Sericin stimulated an overexpression of PPARγ2 in hADSCs-Coll-SS, as compared to hADSCs-Coll, triggering a subsequent upregulated transcription of FAS, aP2 and perilipin markers. As there are no references up to present regarding the effect of natural compound sericin on PPARγ2, FAS, aP2 and perilipin levels of expression, this evaluation also contributes to the novelty of our study. Moreover, based on the expression patterns obtained for these adipogenic markers in both constructs, a higher efficiency of adipogenesis could potentially be correlated with the presence of sericin in the 3D cellular environment.

Taking into consideration all these data, the presence of sericin in the composition of the bioconstruct enhances the bioperformance of the scaffold in terms of proliferation and adipogenesis efficiency. Consequently, the results we obtained *in vitro*, together with the well known sericin stimulatory potential on cellular collagen production, promote Coll-SS biomatrix as a feasible candidate for *in vivo* soft tissue reconstruction and wound healing applications. However, *in vivo* implantation of these constructs should be addressed in further work during ATE applications in order to confirm the reproducibility of these data and to validate safety claims.

## Figures and Tables

**Table 1:** Forward and reverse sequences of primers used to identify early and late adipogenic markers.

Target	Nucleotidic sequence	Fragment length
PPARγ2	F 5′-TTACACAATGCTGGCCTCCTT-3′	99 bp
PPARγ2	R 5′-AGGCTTTCGCAGGCTCTTTAG-3′	

aP2	F 5′-ATGGGATGGAAAATCAACCA-3′	104 bp
aP2	R 5′-GTGGAAGTGACGCCTTTCAT-3′	

Perilipin F	5′-ATGCTTCCAGAAGACCTACA-3′	224 bp
Perilipin R	5′-CAGCTCAGAAGCAATCTTTT-3′	

FAS F	5′-GCTGGAAGTCACCTATGAAG-3′	205 bp
FAS R	5′-TGAAGTCGAAGAAGAAGGAG-3′	

GAPDH F	5′-AAGGTCGGAGTCAACGGATT-3′	224 bp
GAPDH R	5′-CTCCTGGAAGATGGTGATGG-3′	
