# Correction: Dinescu et al. Sericin Enhances the Bioperformance of Collagen-Based Matrices Preseeded with Human-Adipose Derived Stem Cells (hADSCs). *Int. J. Mol. Sci.* 2013, *14*, 1870–1889

**DOI:** 10.3390/ijms262210900

**Published:** 2025-11-10

**Authors:** Sorina Dinescu, Bianca Galateanu, Madalina Albu, Anisoara Cimpean, Anca Dinischiotu, Marieta Costache

**Affiliations:** 1 Department of Biochemistry and Molecular Biology, University of Bucharest, 91–95 Splaiul Independentei, 050095 Bucharest, Romania; sorina_d31@yahoo.com (S.D.); bianca.galateanu@gmail.com (B.G.); anisoara.cimpean@gmail.com (A.C.); dinischiotu@yahoo.com (A.D.); 2 Collagen Department, Leather and Footwear Research Institute, 93, Ion Minulescu, 031215 Bucharest, Romania; albu_mada@yahoo.com

In the original publication [1], there was a mistake in Figure 3 as published. An unintentional error occurred when creating Figure 3: the image corresponding to hADSCs-Coll 14 days was taken from the folder corresponding to hADSCs-Coll-SS 14 days. However, the wrong placing does not affect the results and conclusions of this research. The corrected Figure 3 appears below. The authors state that the scientific conclusions are unaffected. This correction was approved by the Academic Editor. The original publication has also been updated.

**Figure 3:**
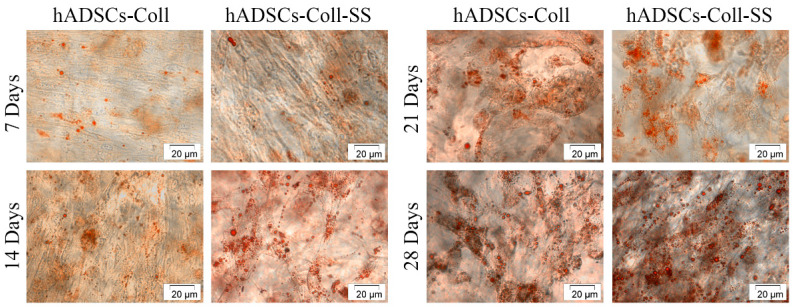
Contrast-phase micrographs of Oil Red O stained hADSCs-Coll and hADSCs-Coll-SS bioconstructs after 7, 14, 21 and 28 days post adipogenic induction.

## References

[1] Dinescu S., Galateanu B., Albu M., Cimpean A., Dinischiotu A., Costache M. (2013). Sericin Enhances the Bioperformance of Collagen-Based Matrices Preseeded with Human-Adipose Derived Stem Cells (hADSCs). Int. J. Mol. Sci..

